# Supplementary figures and images for: Digging deeper: new gene order rearrangements and distinct patterns of codons usage in mitochondrial genomes among shrimps from the Axiidea, Gebiidea and Caridea (Crustacea: Decapoda)
Source: PeerJ. 2017 Mar 1;5:e2982. doi: 10.7717/peerj.2982 (PMC5335691; doi:10.7717/peerj.2982)

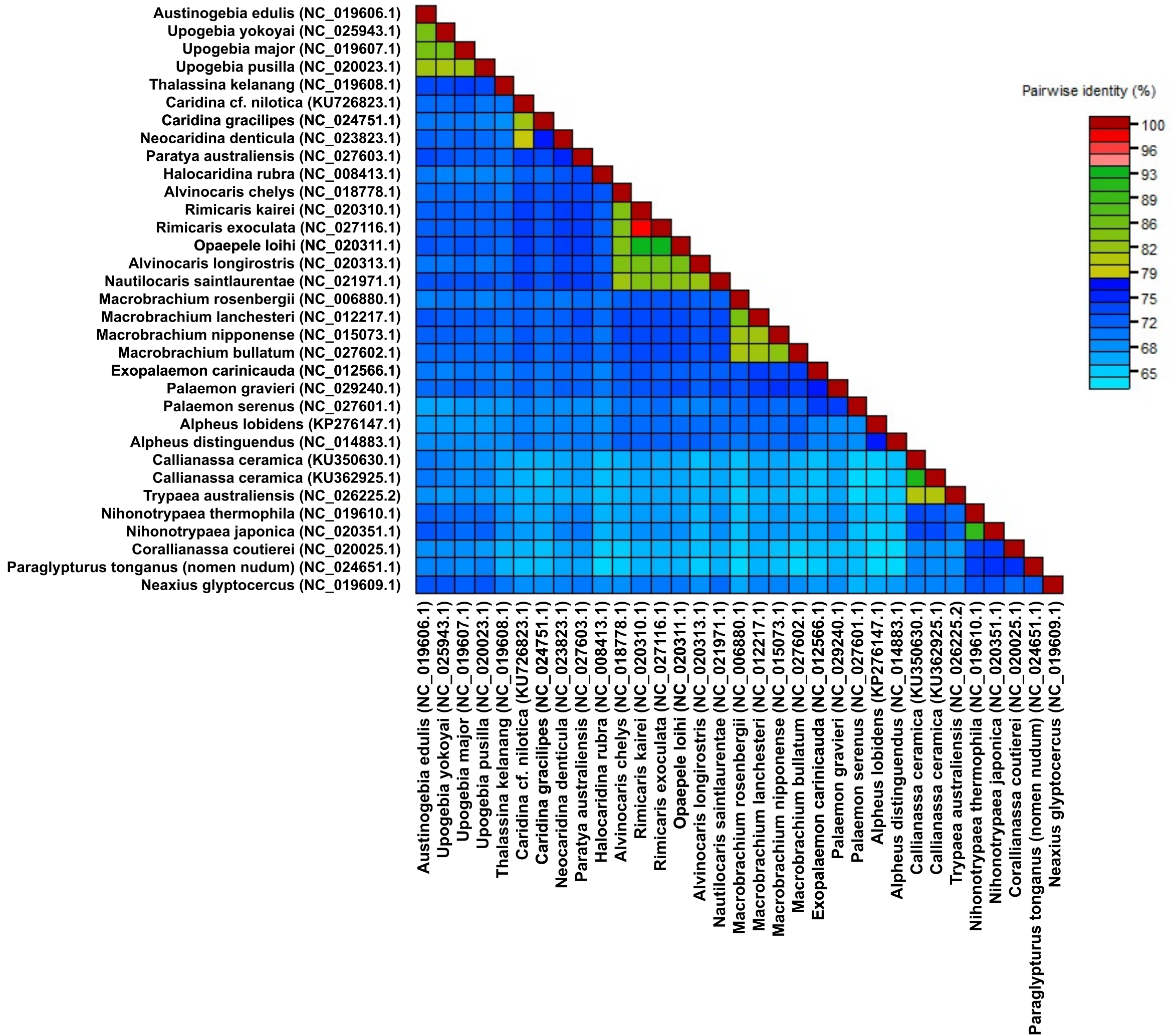

Supplement: Supplemental Information 7 [file peerj-05-2982-s007.pdf]

Ax1 Ax2 Gr

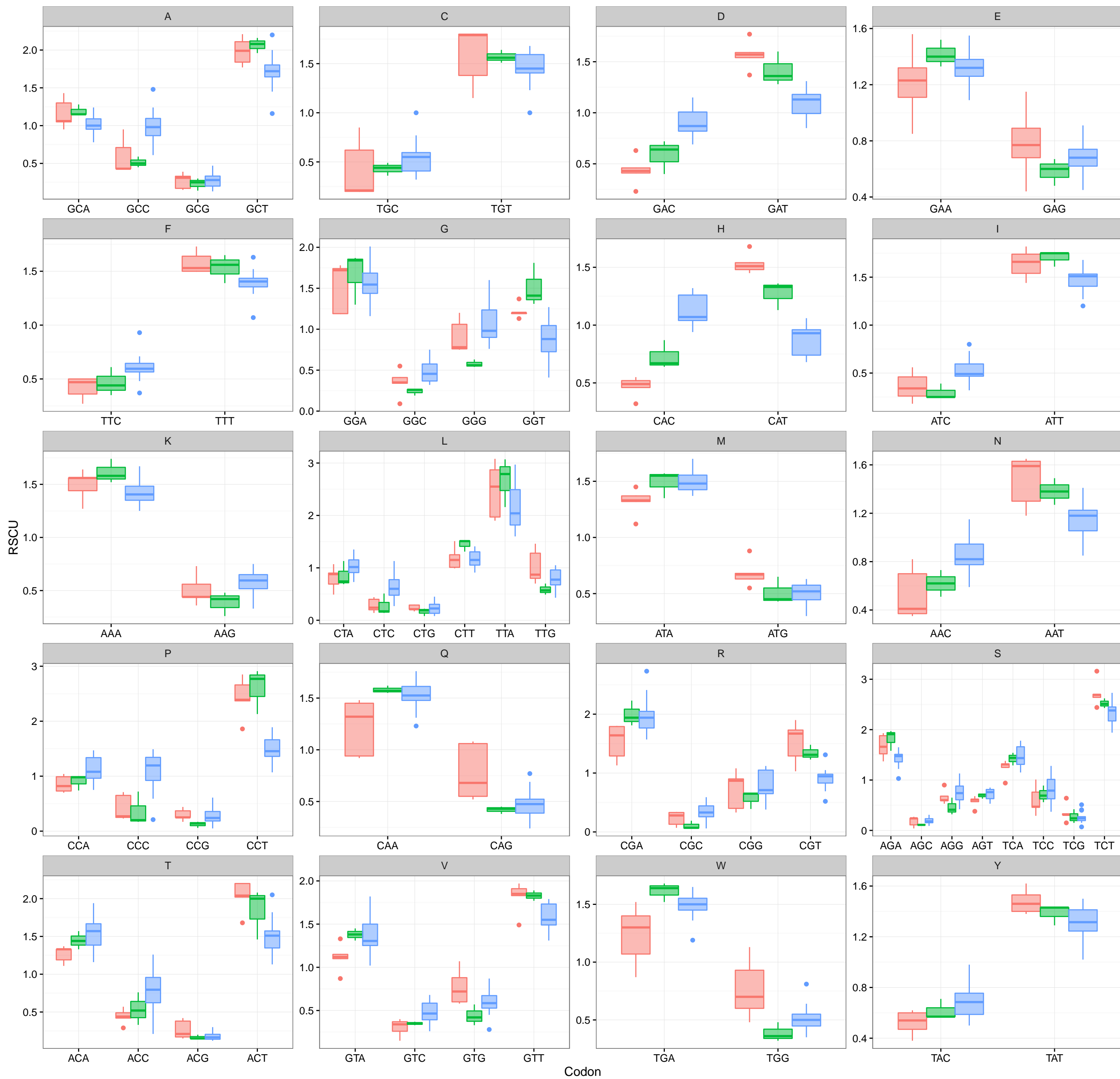

Supplement: Supplemental Information 9 — Encoded amino acid and its corresponding p-value (> or <0.001) is shown at the top of each box plot while synonymous codons are indicated on the x-axis. [file peerj-05-2982-s009.pdf]
