# Supplementary material for: Digging deeper: new gene order rearrangements and distinct patterns of codons usage in mitochondrial genomes among shrimps from the Axiidea, Gebiidea and Caridea (Crustacea: Decapoda)
Source: PeerJ. 2017 Mar 1;5:e2982. doi: 10.7717/peerj.2982 (PMC5335691; doi:10.7717/peerj.2982)

13 PCG (aa) – ML

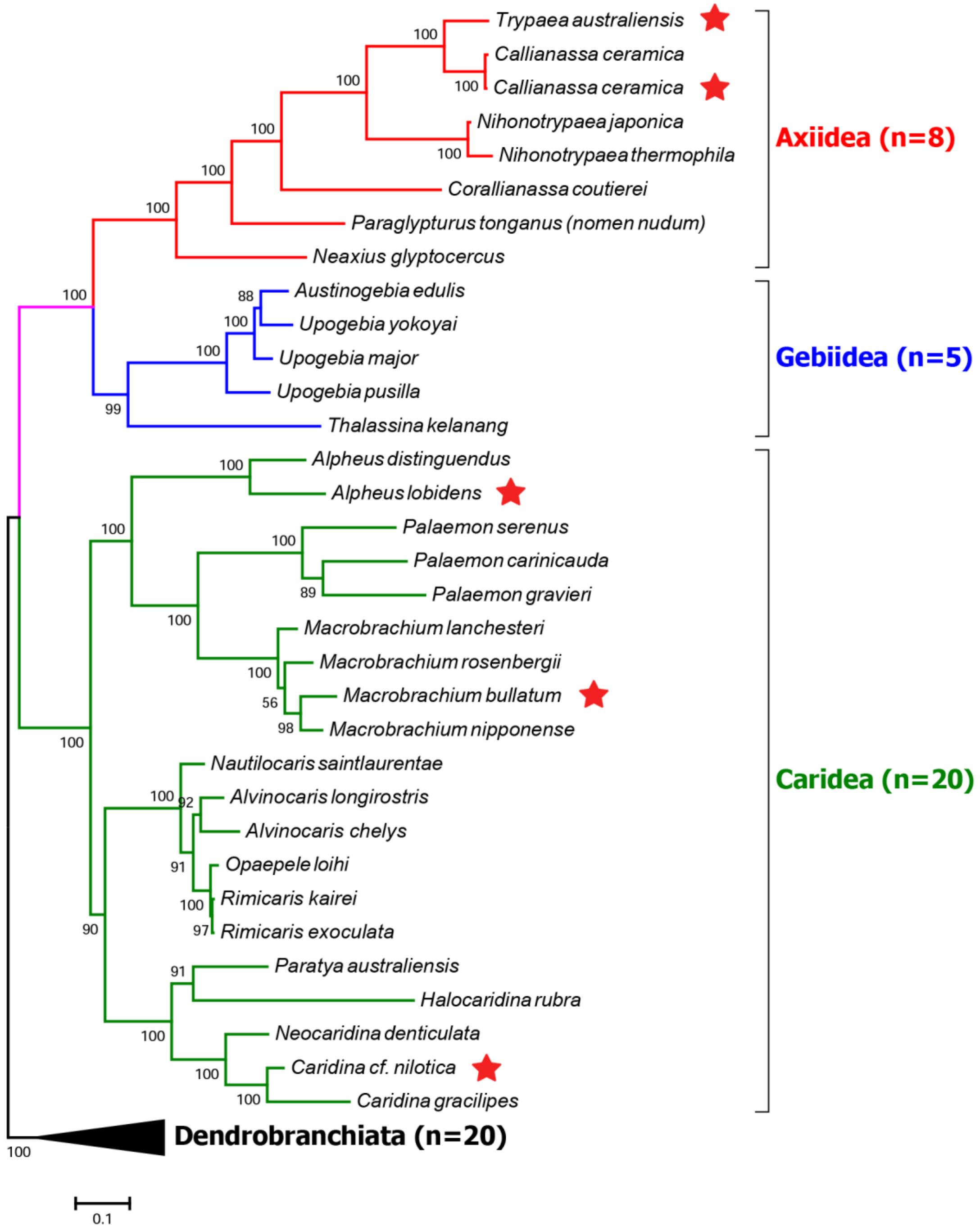

Trypaea australiensis
Callianassa ceramica
Callianassa ceramica
Nihonotrypaea japonica
Nihonotrypaea thermophila
Corallianassa coutierei
Paraglypturus tonganus (nomen nudum)
Neaxius glyptocercus
Austinogebia edulis
Upogebia yokoyai
Upogebia major
Upogebia pusilla
Thalassina kelanang
Alpheus distinguendus
Alpheus lobidens
Palaemon serenus
Palaemon carinicauda
Palaemon gravieri
Macrobrachium lanchesteri
Macrobrachium rosenbergii
Macrobrachium bullatum
Macrobrachium nipponense
Nautilocaris saintlaurentae
Alvinocaris longirostris
Alvinocaris chelys
Opaepele loihi
Rimicaris kairei
Rimicaris exoculata
Paratya australiensis
Halocaridina rubra
Neocaridina denticulata
Caridina cf. nilotica
Caridina gracilipes
Dendrobranchiata (n=20)
Axiidea (n=8)
Gebiidea (n=5)
Caridea (n=20)
0.1

13 PCG (aa) – BI

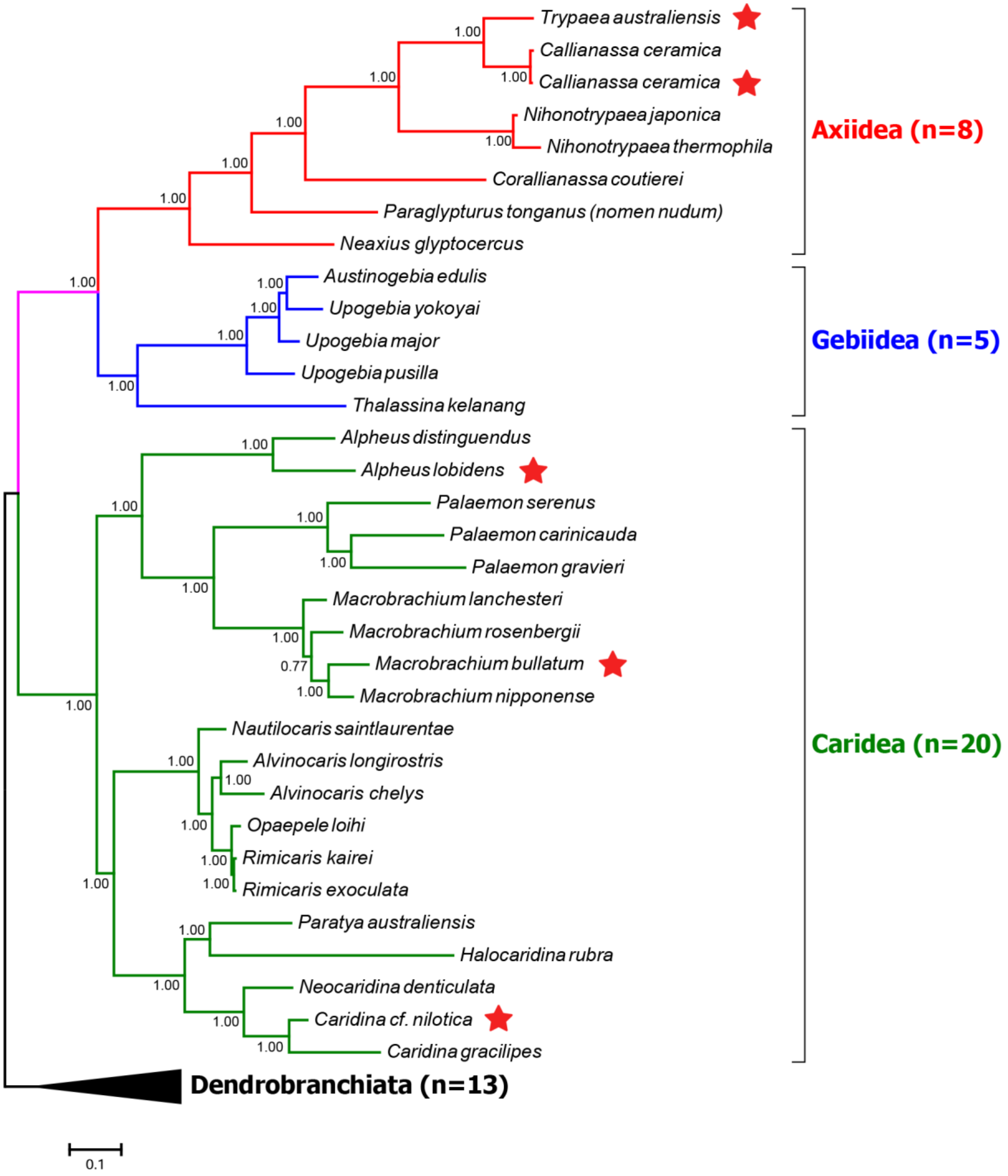
Trypaea australiensis
Callianassa ceramica
Callianassa ceramica
Nihonotrypaea japonica
Nihonotrypaea thermophila
Corallianassa coutierei
Paraglypturus tonganus (nomen nudum)
Neaxius glyptocercus
Austinogebia edulis
Upogebia yokoyai
Upogebia major
Upogebia pusilla
Thalassina kelanang
Alpheus distinguendus
Alpheus lobidens
Palaemon serenus
Palaemon carinicauda
Palaemon gravieri
Macrobrachium lanchesteri
Macrobrachium rosenbergii
Macrobrachium bullatum
Macrobrachium nipponense
Nautilocaris saintlaurentae
Alvinocaris longirostris
Alvinocaris chelys
Opaepele loihi
Rimicaris kairei
Rimicaris exoculata
Paratya australiensis
Halocaridina rubra
Neocaridina denticulata
Caridina cf. nilotica
Caridina gracilipes
Dendrobranchiata (n=13)
Axiidea (n=8)
Gebiidea (n=5)
Caridea (n=20)
0.77
1.00
0.1

13 PCG (nt) – ML

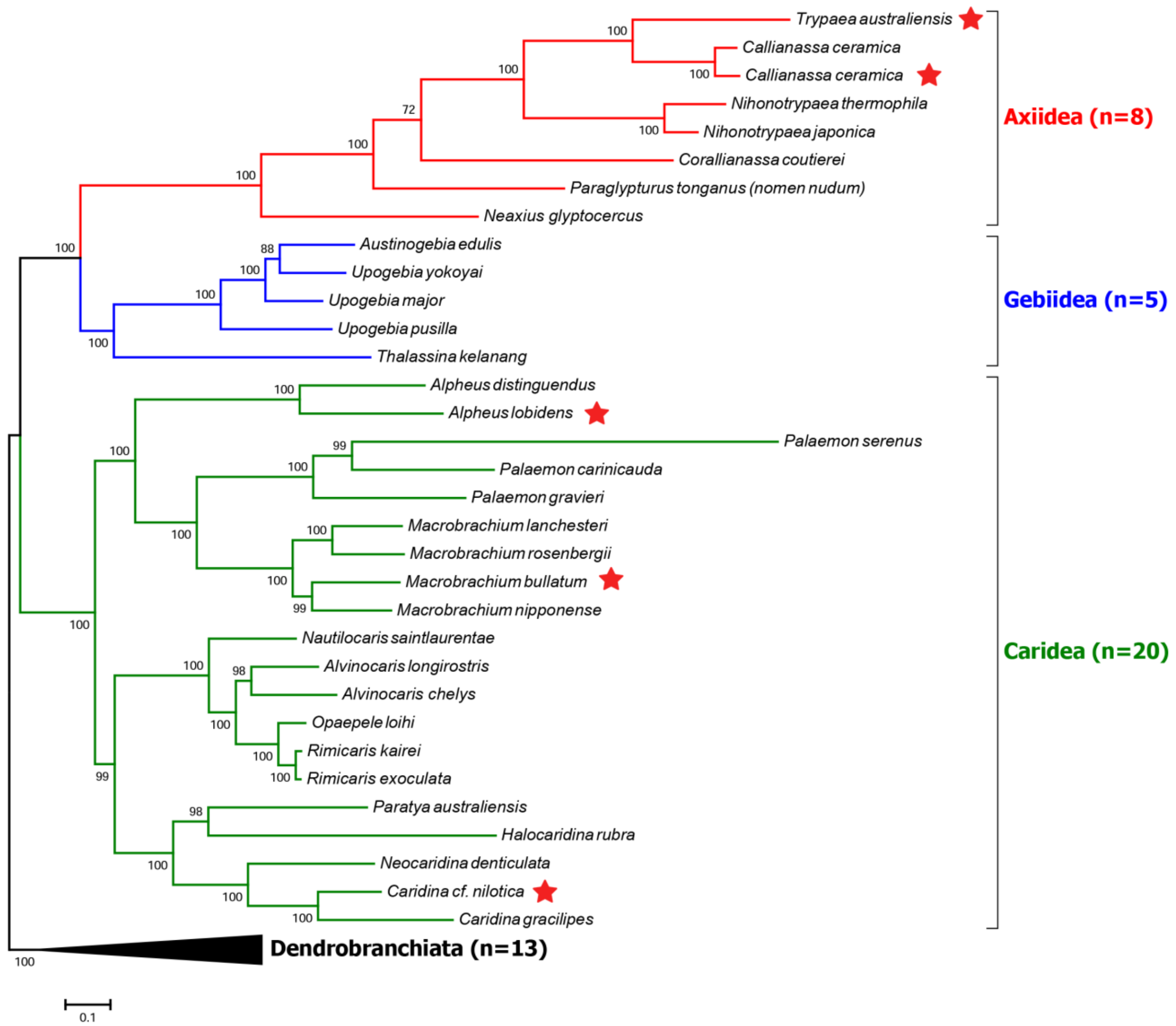

Trypaea australiensis
Callianassa ceramica
Callianassa ceramica
Nihonotrypaea thermophila
Nihonotrypaea japonica
Corallianassa coutierei
Paraglypturus tonganus (nomen nudum)
Neaxius glyptocercus
Axiidea (n=8)
Austinogebia edulis
Upogebia yokoyai
Upogebia major
Upogebia pusilla
Thalassina kelanang
Gebiidea (n=5)
Alpheus distinguendus
Alpheus lobidens
Palaemon serenus
Palaemon carinicauda
Palaemon gravieri
Macrobrachium lanchesteri
Macrobrachium rosenbergii
Macrobrachium bullatum
Macrobrachium nipponense
Nautilocaris saintlaurentae
Alvinocaris longirostris
Alvinocaris chelys
Opaepele loihi
Rimicaris kairei
Rimicaris exoculata
Paratya australiensis
Halocaridina rubra
Neocaridina denticulata
Caridina cf. nilotica
Caridina gracilipes
Caridea (n=20)
Dendrobranchiata (n=13)
0.1

13 PCG (nt) – BI

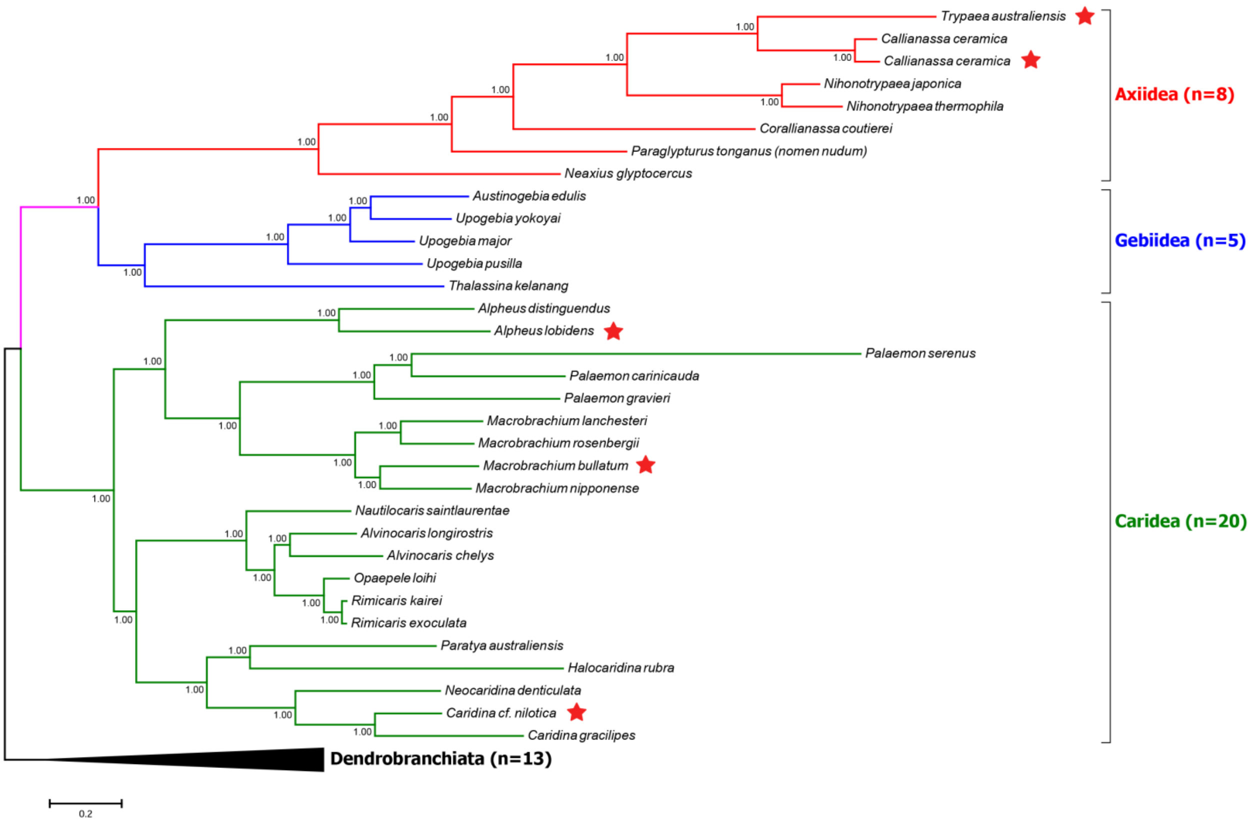

Trypaea australiensis
Callianassa ceramica
Callianassa ceramica
Nihonotrypaea japonica
Nihonotrypaea thermophila
Corallianassa coutierei
Paraglypturus tonganus (nomen nudum)
Neaxius glyptocercus
Austinogebia edulis
Upogebia yokoyai
Upogebia major
Upogebia pusilla
Thalassina kelanang
Alpheus distinguendus
Alpheus lobidens
Palaemon serenus
Palaemon carinicauda
Palaemon gravieri
Macrobrachium lanchesteri
Macrobrachium rosenbergii
Macrobrachium bullatum
Macrobrachium nipponense
Nautilocaris saintlaurentae
Alvinocaris longirostris
Alvinocaris chelys
Opaepele loihi
Rimicaris kairei
Rimicaris exoculata
Paratya australiensis
Halocaridina rubra
Neocaridina denticulata
Caridina cf. nilotica
Caridina gracilipes
Dendrobranchiata (n=13)
Axiidea (n=8)
Gebiidea (n=5)
Caridea (n=20)
1.00
0.2

13 PCG (nt) – partitioned by codon – ML

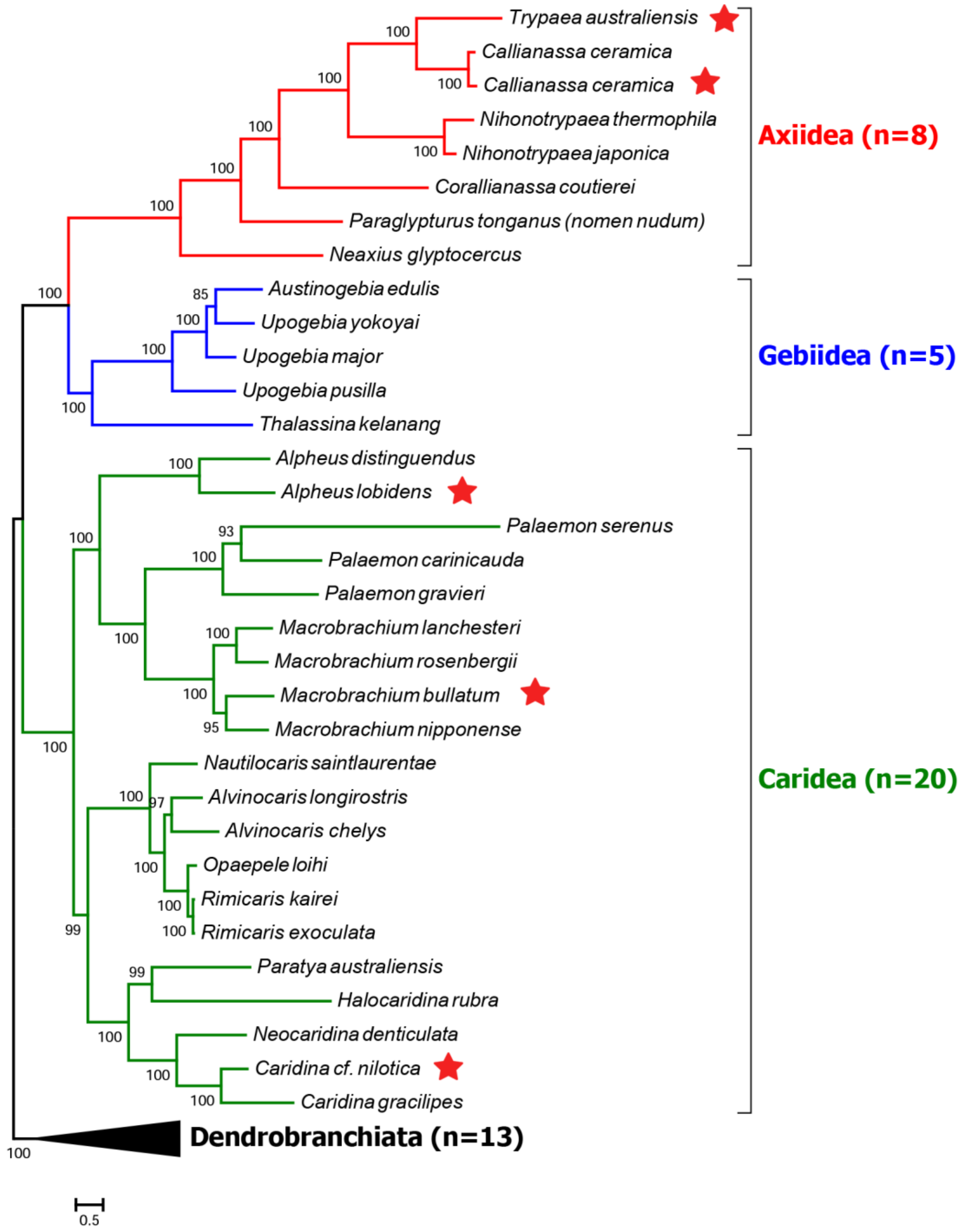

13 PCG (nt) – partitioned by codon – BI

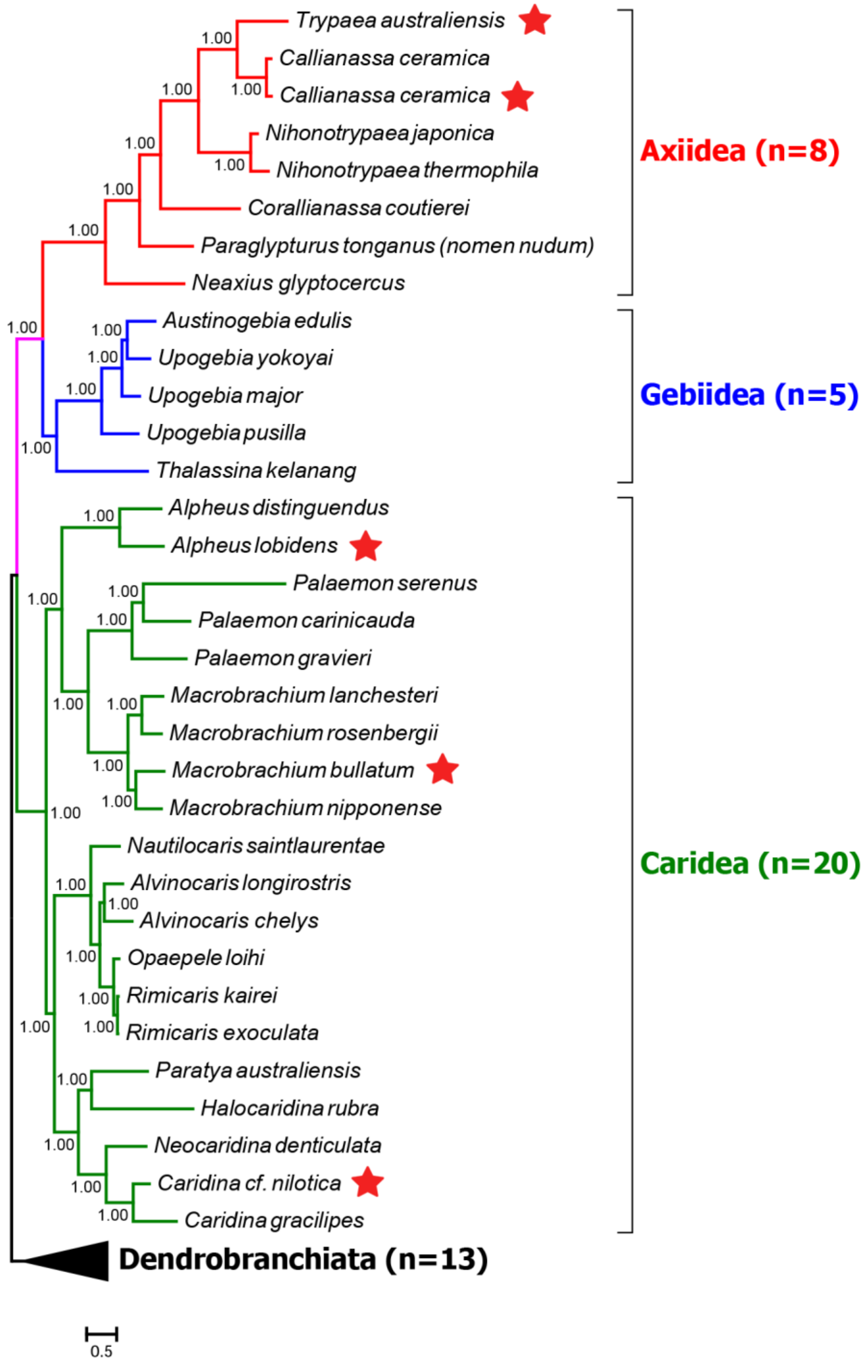

13 PCG (aa) + 12S + 16S – ML

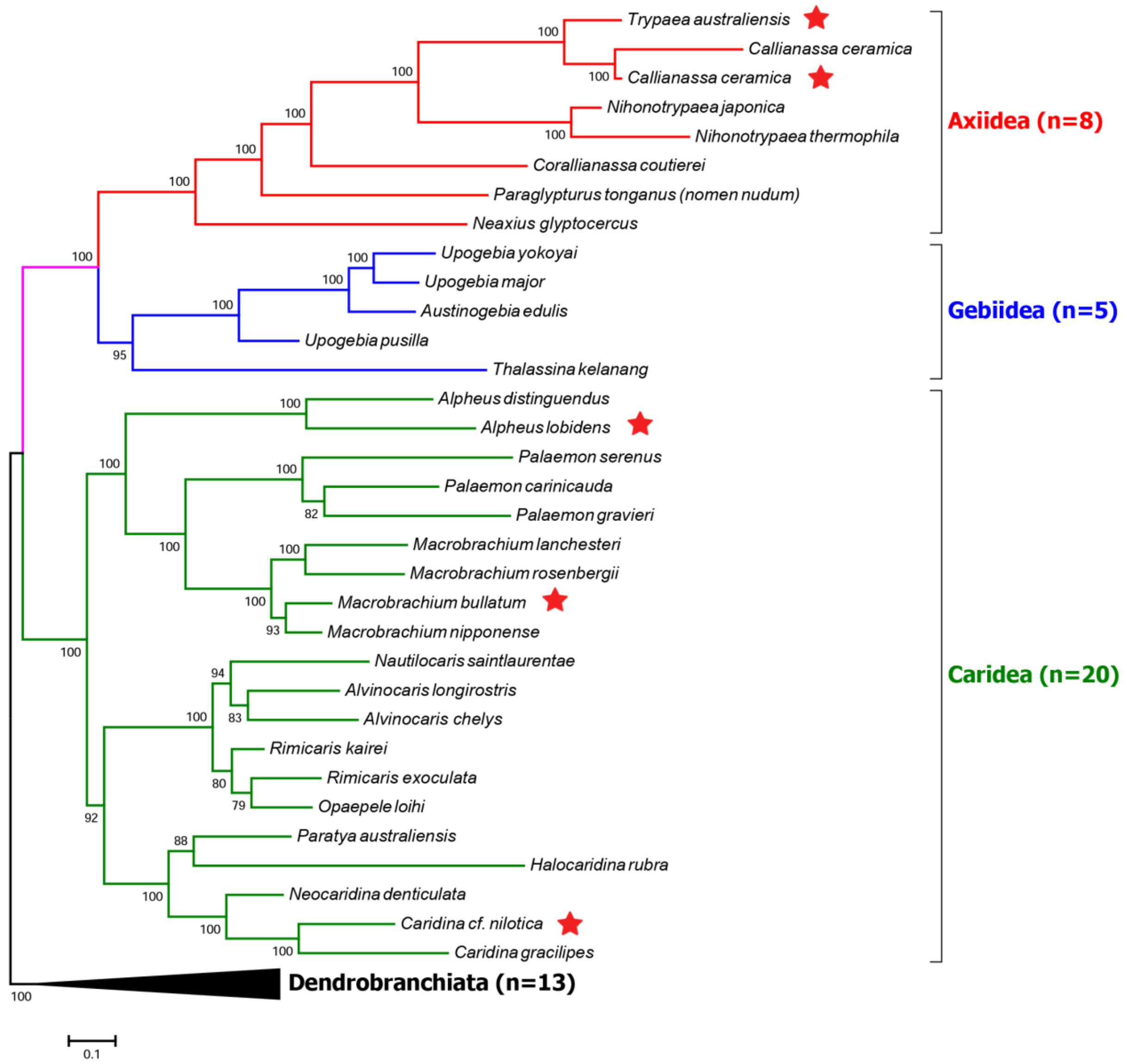

13 PCG (aa) + 12S + 16S – BI

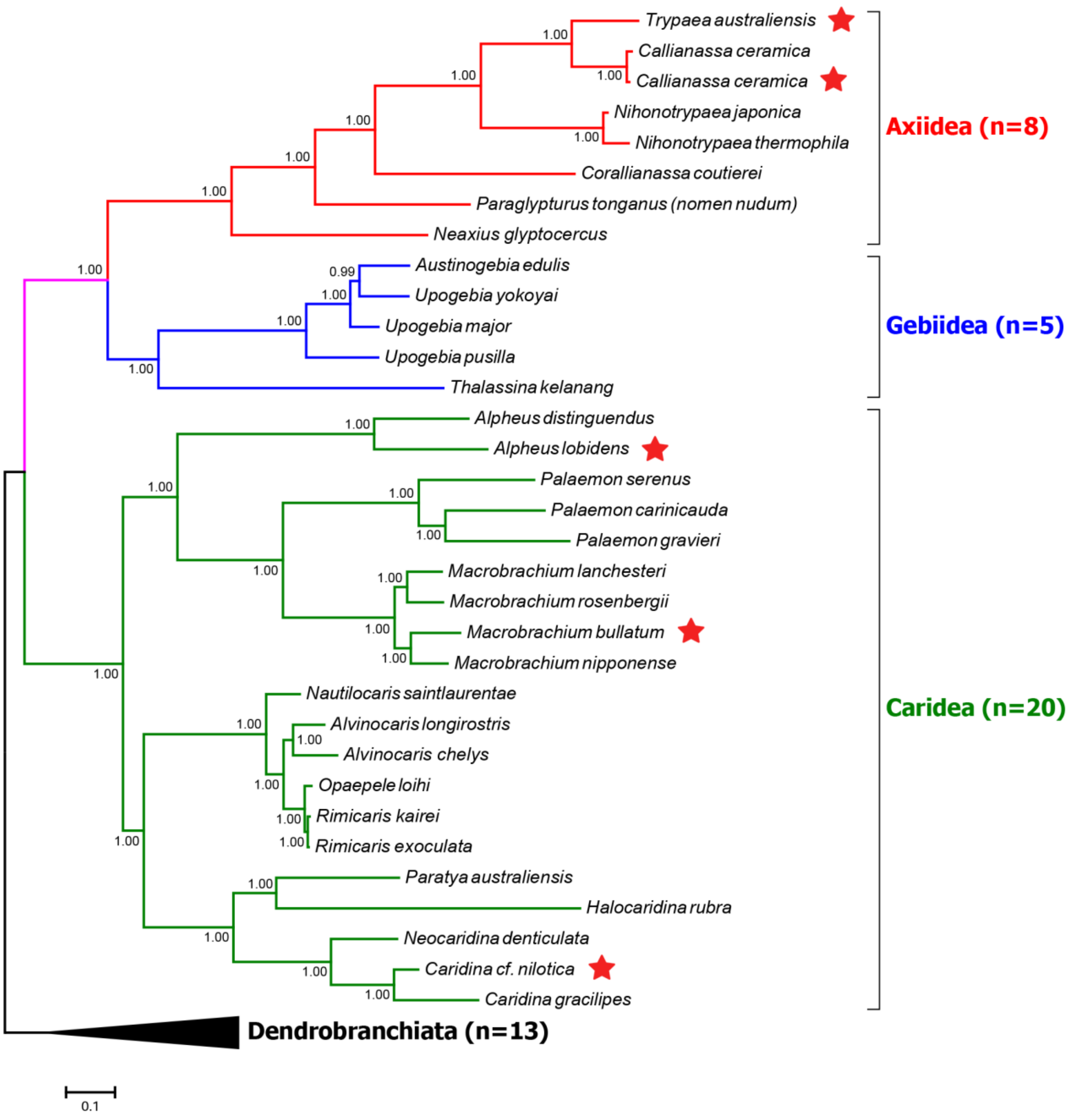

13 PCG (nt) + 12S + 16S – ML

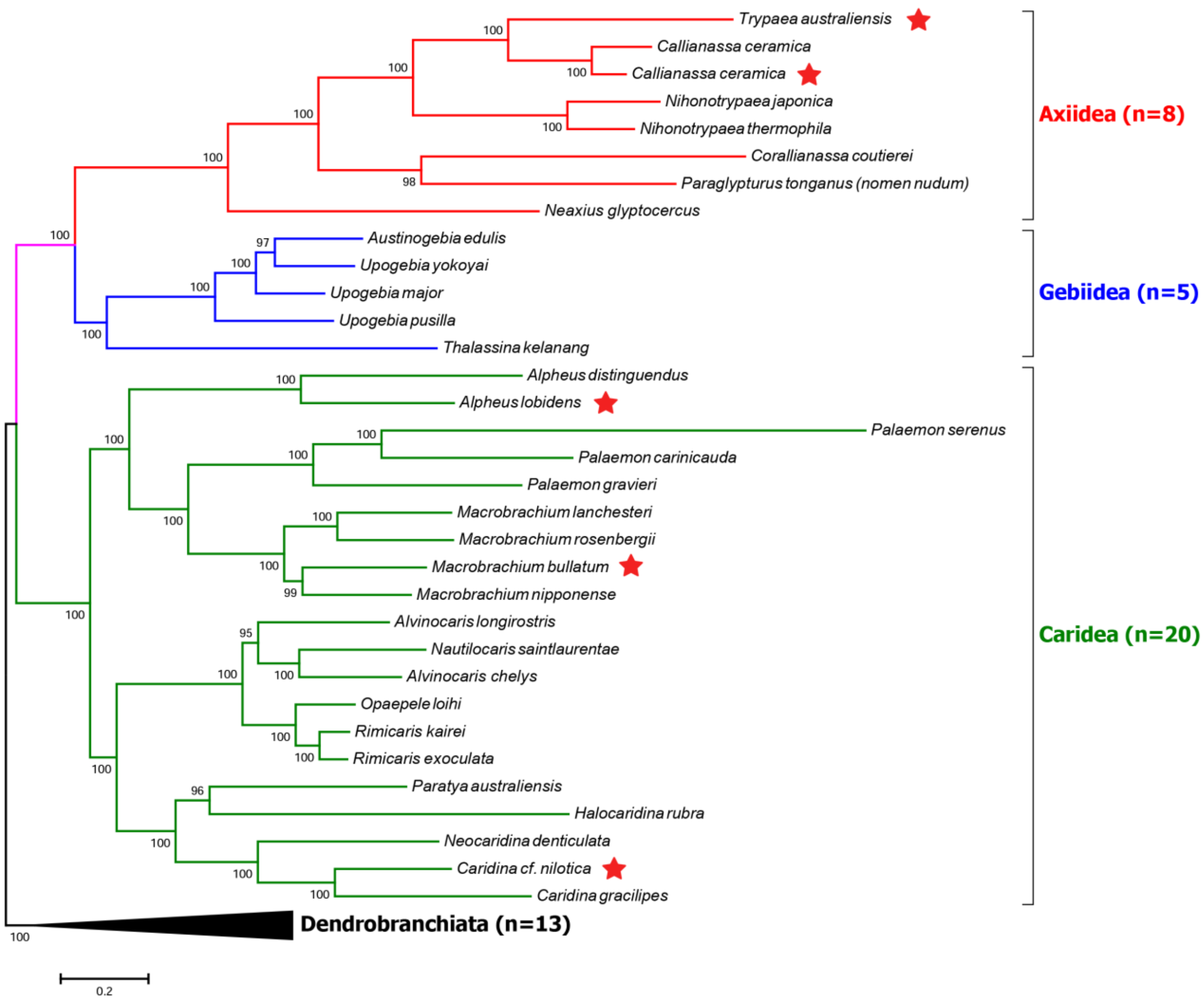

13 PCG (nt) + 12S + 16S – BI

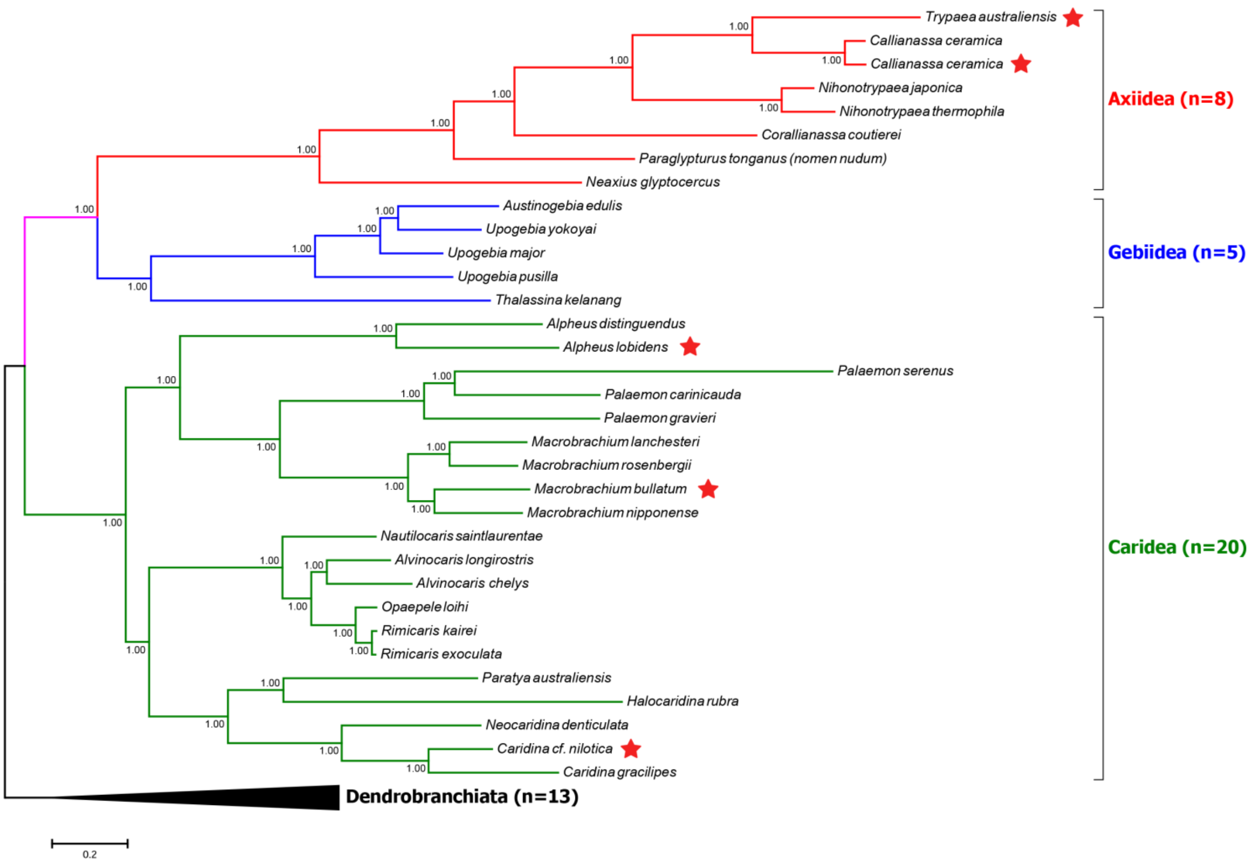

Supplement: Supplemental Information 8 — All trees were constructed using IQ-TREE with optimized partitioning scheme. Trees were rooted with members of Dendrobranchiata as the outgroup. Red stars next to tip labels indicate mitogenomes reported in this study. PCG, protein coding gene; ML, maximum likelihood; aa, amino acid; nt, nucleotide; BI, Bayesian inference. [file peerj-05-2982-s008.pdf]
